# Histone H3 Lysine 9 Acetylation Plays a Role in Adipogenesis of Periodontal Ligament-Derived Stem Cells

**DOI:** 10.3390/epigenomes9020015

**Published:** 2025-05-24

**Authors:** Julio A. Montero-Del-Toro, Angelica A. Serralta-Interian, Geovanny I. Nic-Can, Mónica Lamas, Rodrigo A. Rivera-Solís, Beatriz A. Rodas-Junco

**Affiliations:** 1 Facultad de Ingeniería Química, Universidad Autónoma de Yucatán, Mérida 97203, Mexico; a11003655@alumnos.uady.mx (J.A.M.-D.-T.);; 2 Laboratorio de Células Troncales, Facultad de Odontología, Universidad Autónoma de Yucatán, Mérida 97000, Mexico; 3 SECIHTI-Facultad de Ingeniería Química, Universidad Autónoma de Yucatán, Mérida 97203, Mexico; geovanny.nic@correo.uady.mx; 4 Departamento de Farmacobiología, Centro de Investigación y de Estudios Avanzados-Sede Sur, Ciudad de México 07360, Mexico; mlamas@cinvestav.mx

**Keywords:** dental stem cells, histone deacetylase inhibitors, adipogenic differentiation, periodontal ligament stem cells, histone H3 lysine 9 acetylation

## Abstract

Background: The epigenetic regulation of adipogenic differentiation in dental stem cells (DSCs) remains poorly understood, as research has prioritized osteogenic differentiation for dental applications. However, elucidating these mechanisms could enable novel regenerative strategies for soft tissue engineering. Periodontal ligament stem cells (PDLSCs) exhibit notable adipogenic potential, possibly linked to histone 3 acetylation at lysine 9 (H3K9ac); however, the mechanistic role of this modification remains unclear. Methods: To address this gap, we investigated how histone deacetylase inhibitors (HDACis)—valproic acid (VPA, 8 mM) and trichostatin A (TSA, 100 nM)—modulate H3K9ac dynamics, adipogenic gene expression (*C/EBPβ* and *PPARγ-2*), and chromatin remodeling during PDLSCs differentiation. Techniques used included quantitative PCR (qPCR), lipid droplet analysis, and chromatin immunoprecipitation followed by qPCR (ChIP-qPCR). Results: TSA-treated cells exhibited increased lipid deposition with smaller lipid droplets compared to VPA-treated cells. Global H3K9ac levels correlated positively with adipogenic progression. VPA induced early upregulation of *C/EBPβ* and *PPARγ-2* (day 7), whereas TSA triggered a delayed but stronger *PPARγ-2* expression. ChIP-qPCR analysis revealed significant H3K9ac enrichment at the *PPARγ-2* promoter in TSA-treated cells, indicating enhanced chromatin accessibility. Conclusions: These findings demonstrate that H3K9ac-mediated epigenetic remodeling plays a critical role in the adipogenic differentiation of PDLSCs and identifies TSA as a potential tool for modulating this process.

## 1. Introduction

Mesenchymal stem cells (MSCs) represent a heterogeneous population with multilineage differentiation potential, including the adipogenic lineage [1,2]. Adipogenic differentiation is tightly regulated by a complex network of genetic and epigenetic factors [3,4]. At the genetic level, transcription factors (TFs) such as the peroxisome proliferator-activated receptor-γ (PPARγ)—considered a master regulator—and members of the CCAAT/enhancer-binding protein (C/EBP) family, particularly C/EBPα and C/EBPβ, act synergistically through positive feedback loops to sequentially activate gene programs involved in adipocyte maturation [5,6,7]. Concurrently, epigenetic mechanisms dynamically modulate chromatin architecture, regulating gene accessibility throughout the differentiation process [8,9,10].

Although adipogenesis has been extensively studied in conventional models such as 3T3-L1 preadipocytes [11,12] and MSCs derived from bone marrow or adipose tissue [13,14], recent research has shifted focus toward alternative cell sources with adipogenic potential. Among these, dental stem cells (DSCs) have emerged as a promising model for exploring novel aspects of adipogenic differentiation [15,16]. Derived from ectomesenchymal tissues, as the dental pulp stem cells (DPSCs), periodontal ligament stem cells (PDLSCs), and dental follicle stem cells (DFSCs), share many characteristics with other MSCs while exhibiting unique differentiation capabilities [15,17,18]. Their clinical accessibility—obtainable through minimally invasive procedures—and remarkable plasticity position DSCs as ideal candidates for investigating regulatory mechanisms of adipogenesis that may remain undetected in conventional models.

The epigenetic regulation of DSCs during differentiation has been studied in various contexts [19,20]; however, evidence specific to adipogenesis remains limited. Nonetheless, recent studies have begun to elucidate potential underlying mechanisms. For instance, Argaez-Sosa et al. [21] demonstrated that DNA methylation patterns at the regulatory regions of *PPARγ-2* correlate with the adipogenic potential of DPSCs. Complementarily, Balam-Lara, et al. [22] reported that overexpression of TEN-ELEVEN TRANSLOCATION 2 (TET2), a methyl cytosine dioxygenase, enhances adipogenic commitment in DPSCs by modulating the transcriptional activity of adipogenic markers such as *PPARγ*, *adiponectin* (ADIPOQ), fatty acid binding protein 4 (FABP4), and *lipoprotein* (LPL). However, these advances focus primarily on DNA methylation, while the role of histone modifications in the adipogenic differentiation of DSCs remains largely underexplored.

This knowledge gap is particularly significant given that histone acetylation—especially at lysine 9 of histone H3 (H3K9ac)—is a key epigenetic mechanism involved in transcriptional activation and cellular plasticity [23,24]. H3K9ac is especially intriguing in the context of DSCs due to its potential to explain the variations in adipogenic capacity observed among different DSC subtypes. Unlike DNA methylation, which is typically associated with stable gene silencing, histone acetylation is dynamic and reversible, offering a more flexible and adaptable regulatory mechanism suited to the physiological demands of ectomesenchymal tissues. This regulatory plasticity positions H3K9ac as a central element in understanding DSC-specific differentiation programs and opens new avenues for its targeted manipulation in regenerative medicine.

The dynamic regulation of H3K9ac becomes especially relevant considering that histone acetylation is finely controlled by the balanced activities of histone acetyltransferases (HATs) and histone deacetylases (HDACs). HDAC inhibitors (HDACis), particularly trichostatin A (TSA) and valproic acid (VPA), have emerged as valuable tools for studying the role of histone acetylation in chromatin remodeling during cell differentiation [25,26]. Evidence indicates that these compounds modulate adipogenesis in a model-specific manner. For example, in murine 3T3-L1 preadipocytes, VPA affects both the initiation and early maturation stages of adipogenesis [27], whereas TSA influences *PPARγ* expression and lipid accumulation through pathways such as AMPK signaling [28]. In the C3H/10T1/2 model, the specific inhibitor MS-275 not only enhances white adipocyte functionality but also induces trans differentiation into a brown-like phenotype [29]. Additionally, dynamic changes in histone marks, including H3K9ac, have been observed throughout the adipogenic differentiation process in adipose-derived MSCs [30].

Recent evidence indicates that HDACis such as TSA and VPA can promote adipogenic differentiation in PDLSCs by increasing H3K9 acetylation and activating key adipogenic genes [31,32]. However, comprehensive analyses of global H3K9ac changes—and their relationship with adipogenic regulators like PPARγ and C/EBPβ during HDACi treatment in PDLSCs—are still lacking. Therefore, this study aimed to evaluate the effects of the HDACis VPA and TSA on PDLSCs to determine how these epidrugs influence global H3K9ac levels throughout adipogenesis, and how this epigenetic modification regulates the expression of adipogenic genes. The findings from this research will not only deepen our understanding of the epigenetic mechanisms that govern DSC plasticity but may also identify novel therapeutic targets for the precise modulation of adipogenesis in regenerative medicine.

## 2. Results

### 2.1. Characterization of Periodontal Ligament Cells

**Figure 1:**
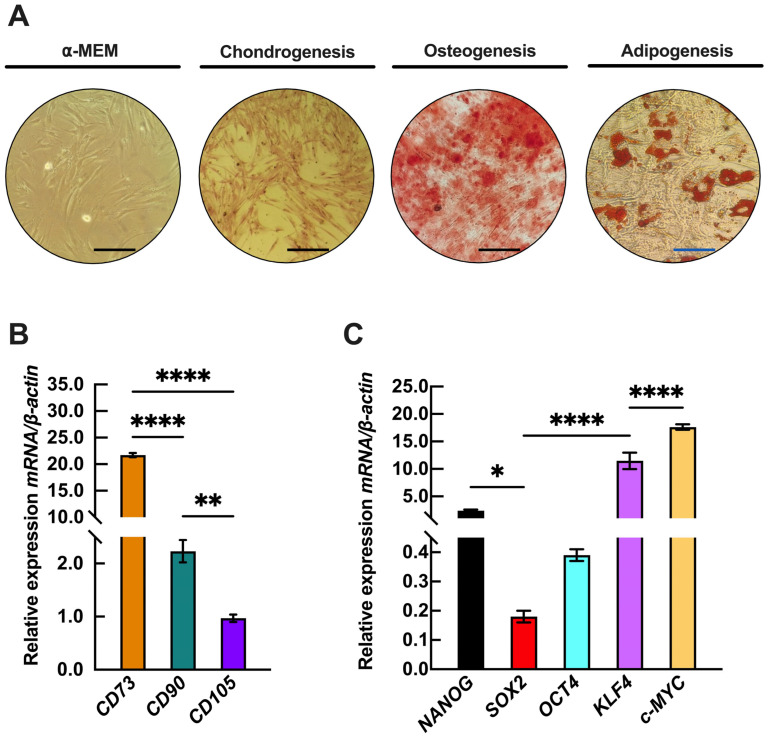
Characterization of mesenchymal properties in periodontal ligament-derived cells. (**A**) Multilineage differentiation potential was assessed on day 14 for chondrogenic and osteogenic lineages, and on day 21 for the adipogenic lineage. (**B**) Expression of mesenchymal surface markers (*CD73*, *CD90*, *CD105*) was analyzed by RT-qPCR. (**C**) Expression profiles of stemness-related markers (*Nanog*, *Sox2*, *Oct4*, *KLF4*, and *c-MYC*) were evaluated by RT-qPCR. All mRNA expression data are presented as mean ± SD (*n* = 3) and normalized to *β-actin* as the housekeeping gene. Statistical significance was determined by one-way ANOVA followed by Tukey’s post hoc test, comparing data sets from the same time point. Significance levels are indicated as * (*p* < 0.05), ** (*p* < 0.01), and **** (*p* < 0.0001). The black and blue scale bars indicate 133 μm and 75 μm, respectively.

Human periodontal ligament cell (PDLC) cultures exhibited an elongated, fibroblast-like spindle shape and demonstrated plasticity toward chondrogenic, osteogenic, and adipogenic lineages (Figure 1A). After 14 days of chondrogenic and osteogenic induction, Alcian blue and Alizarin red staining confirmed the formation of chondrogenic nodules and calcium deposits, respectively. Adipogenic differentiation was evident after 21 days, as lipid droplets were observed under the microscope (Figure 1A). These findings indicate that PDLCs possess multilineage differentiation potential, consistent with the criteria established by the International Society for Stem Cell Research [33]. To further characterize the mesenchymal phenotype of PDLCs, we analyzed the expression of surface markers *CD73*, *CD90*, and *CD105*, finding that *CD73* was expressed at higher levels compared to *CD90* and *CD105* (Figure 1B). Additionally, we evaluated the expression of key embryonic stem cell markers associated with self-renewal and pluripotency, including *NANOG*, *SOX2*, *OCT4*, *KLF4*, and *c-MYC*. As shown in Figure 1C, *NANOG*, *OCT4*, *KLF4*, and *c-MYC* exhibited the highest expression levels, whereas *SOX2* exhibited the lowest. These findings confirm that PDLCs possess stem cell characteristics and maintain a plastic state, supporting their potential for multilineage differentiation.

### 2.2. VPA and TSA Assays Improve the Adipogenic Capacity of PDLSCs

**Figure 2:**
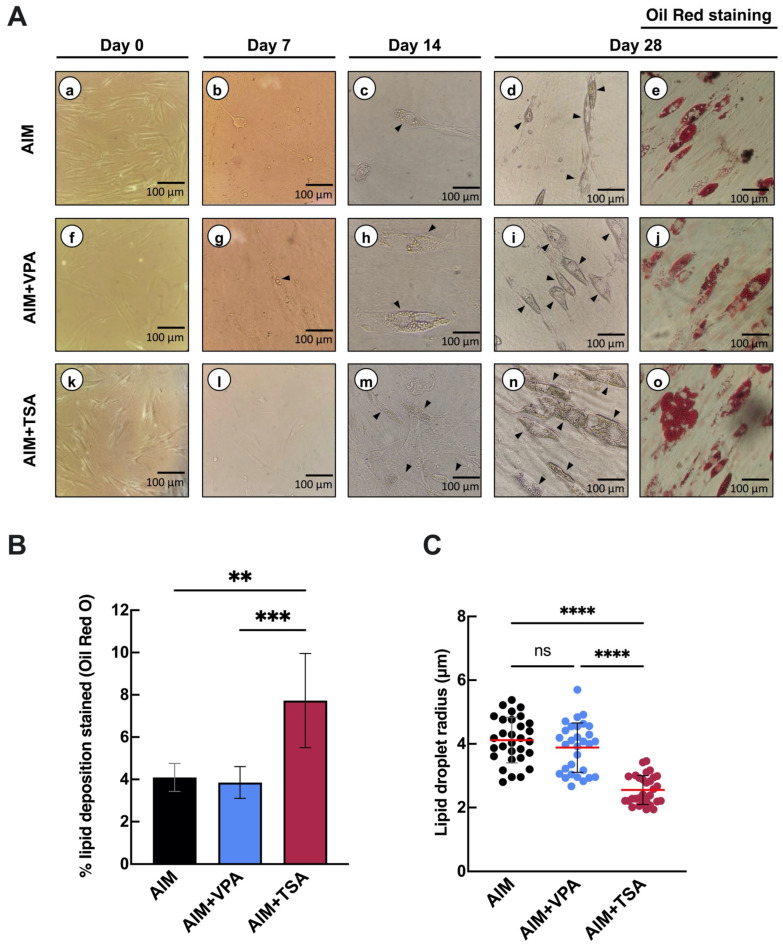
Effect of histone deacetylase inhibitors on adipogenic induction in periodontal ligament stem cells. (**A**) Morphological changes during 28-day adipogenic induction. Panels (**a**–**e**) show cultures treated with adipogenic induction medium only (AIM), (**f**–**j**) AIM supplemented with valproic acid (AIM + VPA), and (**k**–**o**) AIM supplemented with TSA (AIM + TSA). All groups were maintained in AIM and basal α-MEM medium (α-MEM). Panels (**e**,**j**,**o**) display Oil Red O staining of lipid droplets on day 28 of adipogenic induction. Photomicrographs (20×) depict progressive differentiation, with arrows (►) highlighting key morphological changes. (**B**) Quantification of lipid accumulation on day 28, expressed as the percentage of Oil Red O-positive area (red pixels/total pixels) in representative fields (*n* = 30; image resolution: 4080 × 3072 pixels). (**C**) Average radius of lipid droplets measured after 28 days of adipogenic induction (*n* = 30). Statistical significance was determined by one-way ANOVA followed by Tukey’s post hoc test, comparing data sets from the same time point. Significance levels are indicated as ** (*p* < 0.01), *** (*p* < 0.001), and **** (*p* < 0.0001). ns: not significant.

To investigate whether VPA or TSA affects the adipogenic differentiation of PDLSCs, cells were divided into three groups: a control group cultured in the adipogenic induction medium (AIM) without inhibitors, and two experimental groups pretreated with either VPA (8 mM) or TSA (100 nM) for 72 h prior to adipogenic induction with AIM. In control cultures, numerous intracellular lipid droplets were observed by day 14 (Figure 2A-c). In contrast, cells treated with the inhibitors exhibited distinct morphological changes depending on the treatment. VPA-treated cells demonstrated early lipid accumulation, detectable by day 7 (Figure 2A-g), with a progressive increase in adipocyte numbers observed by day 28 (Figure 2A-i,j). In TSA-treated cells, intracellular lipids appeared later, around day 14 (Figure 2A-m); however, by day 28, these cells exhibited a higher density of lipid droplets compared to both the control and VPA-treated groups (Figure 2A-n,o). Notably, TSA-treated preadipocytes were smaller and more elongated than those in the control group (Figure 2A-e,o). To quantitatively assess lipid accumulation, intracellular lipid deposition per unit area and lipid droplet radius were analyzed using ImageJ v.1.52 software. Oil Red O staining revealed that TSA-treated cells exhibited a twofold increase in lipid deposition compared to VPA-treated and control cells (Figure 2B). Additionally, analysis of lipid droplet size showed that TSA-treated cells formed smaller lipid droplets (2.55 ± 0.45 μm radius) compared to VPA-treated (3.88 ± 0.78 μm) and control cells (4.12 ± 0.72 μm) (Figure 2C). Overall, these results suggest that epigenetic inhibitors promote adipogenic differentiation in PDLSCs through distinct mechanisms, with VPA inducing early adipogenic differentiation, while TSA enhances long-term lipid accumulation.

### 2.3. Effect of HDAC Inhibitors on H3K9 Acetylation Levels During Adipogenesis

To determine whether the phenotypic changes observed during adipogenesis in the presence of VPA and TSA are attributable not only to the induction medium but also to HDAC inhibition, we analyzed H3K9 acetylation levels in PDLSCs using Western blotting.

**Figure 3:**
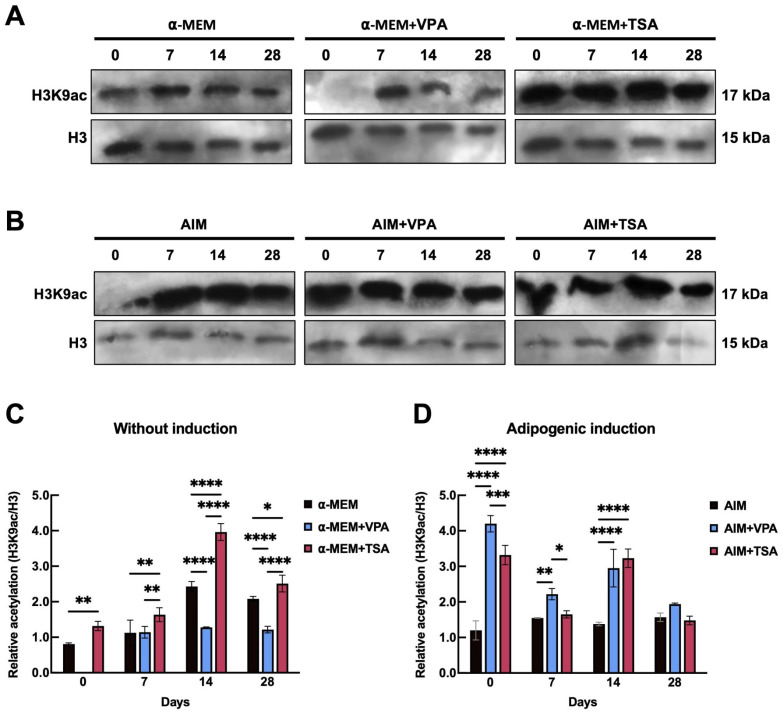
Dynamics of H3K9 acetylation mediated by VPA or TSA in periodontal ligament stem cells. (**A**) Western blot analysis of H3K9ac (17 kDa) and total H3 (15 kDa) following a 72-h pretreatment with 8 mM VPA or 100 nM TSA, with cells subsequently maintained in α-MEM basal medium for 28 days. (**B**) Quantitative analysis of H3K9ac levels normalized to total H3 from panel A. (**C**) Western blot analysis of H3K9ac and total H3 in cells pretreated with VPA or TSA for 72 h and subsequently maintained in adipogenic induction medium (AIM) for 28 days. (**D**) Quantification of H3K9ac/H3 ratios from panel C. For all Western blot analyses, 10 μg of protein was loaded per lane. Data represent mean ± SD (*n* = 3). Statistical significance was determined by two-way ANOVA followed by Tukey’s post hoc test, comparing time points within each treatment group. Significance levels are indicated as follows: ns (not significant, *p* ≥ 0.05), * *p* < 0.05, ** *p* < 0.01, *** *p* < 0.001, and **** *p* < 0.0001.

Protein samples were collected from both undifferentiated and differentiated PDLSCs on days 0, 7, 14, and 28 (Figure 3). In TSA-treated cells, H3K9ac levels increased beginning at day 7 and reached their peak at day 14. Interestingly, in VPA-treated cells, an increase in H3K9ac levels was detected only at day 7 (Figure 3A,B). These findings suggest that the effects of HDACis are most pronounced shortly after exposure, promoting increased chromatin accessibility. Notably, in cells induced to undergo adipogenesis, H3K9ac levels were higher than in undifferentiated cells, indicating a synergistic effect between the induction medium and the inhibitors (Figure 3C,D). In this context, both VPA and TSA showed acetylation peaks on days 0 and 14, which declined by day 28. These peaks coincided with morphological changes characteristic of adipocyte development, such as lipid droplet formation. Densitometric analysis revealed that on day 0, TSA-treated cells exhibited approximately 25% higher H3K9ac levels compared to VPA-treated cells (Figure 3D). By day 7, acetylation levels decreased by approximately 10% in both VPA- and TSA-treated cells compared to the control. However, by day 14, treatment with either inhibitor resulted in a twofold increase in H3K9ac levels relative to the control. By day 28, acetylation levels were similar across all conditions (Figure 3C,D). These results confirm that HDACis promote H3K9 acetylation, exerting a positive influence on adipogenic differentiation. The temporal correlation between peaks in H3K9ac and morphological changes, such as lipid droplet formation, further suggests that this epigenetic modification plays a critical role in PDLSC adipogenesis.

### 2.4. Effect of TSA and VPA on PPARγ-2 and C/EBPβ Gene Expression

**Figure 4:**
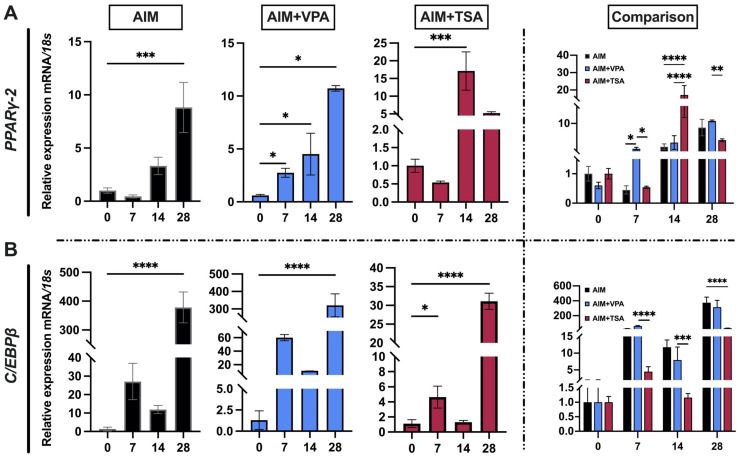
Analysis of *PPARγ-2* and *C/EBPβ* expression during adipogenic differentiation of periodontal ligament stem cells: Periodontal ligament stem cells were pretreated with 8 mM VPA or 100 nM TSA for 72 h, followed by a 28-day adipogenic induction using adipogenic induction medium (AIM). Cells were harvested at specified time points (days 0, 7, 14, and 28) for gene expression analysis. (**A**) *PPARγ-2* and (**B**) *C/EBPβ* mRNA levels were quantified by RT-qPCR, with expression normalized to the *18S rRNA* housekeeping gene. Data represent the mean ± SD of three independent experiments (*n* = 3). Statistical significance was determined by one-way ANOVA with Dunnett’s post hoc test, comparing each time point to day 0 controls. Significance levels are indicated as: * *p* < 0.05, ** *p* < 0.01, *** *p* < 0.001, and **** *p* < 0.0001.

To investigate the impact of HDAC inhibition on adipogenic gene expression, we analyzed the transcriptional levels of *PPARγ-2* and *C/EBPβ*, key TFs involved in early adipocyte differentiation. The results showed a progressive increase in both *PPARγ-2* and *C/EBPβ* regardless of treatment, though there were distinct differences in the timing and magnitude of their responses. In the control group, cells cultured in adipogenic medium alone exhibited a gradual increase in *PPARγ-2* expression, reaching an eight-fold increase compared to day 0. In contrast, VPA-treated cells demonstrated an early response, with a significant 5-fold increase in *PPARγ-2* transcript levels by day 7 (Figure 4A). Meanwhile, TSA-treated cells exhibited a delayed but more pronounced response, reaching their highest expression levels on day 14, with a 20-fold increase compared to day 0 (Figure 4A). Regarding *C/EBPβ*, a similar pattern was observed for both treatments, with expression peaks occurring on days 7 and 28 of adipogenic induction (Figure 4B). VPA-treated cells exhibited the highest expression levels, with a 150-fold increase in *C/EBPβ* on day 7 compared to day 0. In contrast, TSA-treated cells demonstrated a more gradual upregulation, reaching a 15-fold increase by day 28 (Figure 4B). These findings suggest that HDACis likely prevent the formation of transcriptional repression complexes, in which HDACs play a central role, thereby promoting histone acetylation. This, in turn, enhances the transcription of *PPARγ-2* and *C/EBPβ* during adipogenesis in cells treated with VPA and TSA.

### 2.5. Analysis of H3K9ac Enrichment in PPARγ-2 During Adipogenesis

**Figure 5:**
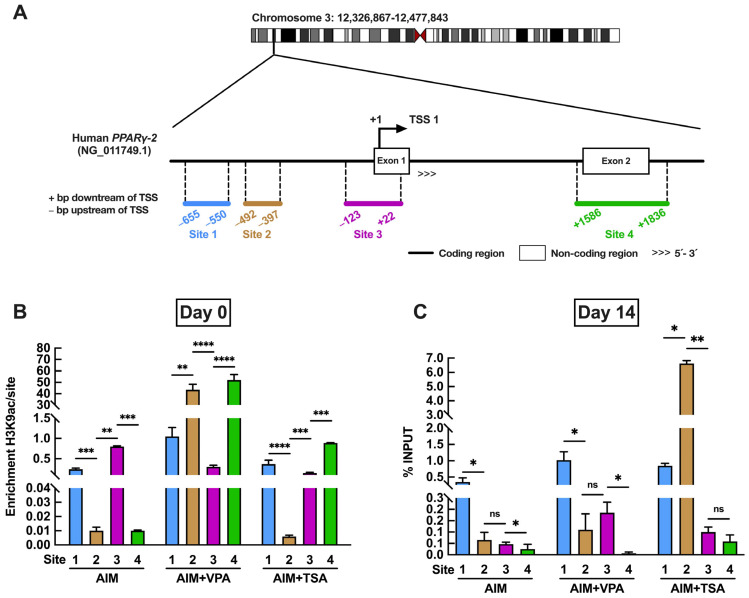
H3K9ac enrichment profile at the PPARγ-2 promoter region during adipogenesis in periodontal ligament stem cells. (**A**) Genomic map showing predicted binding sites within the *PPARγ-2* promoter region. (**B**) H3K9ac enrichment at *PPARγ-2* promoter binding sites following treatment with VPA or TSA on day 0 of adipogenic induction. (**C**) H3K9ac enrichment at putative *PPARγ-2* promoter (Prom-*PPARγ2*) binding sites after VPA or TSA treatment on day 14 of adipogenic induction. Data represent mean ± SD (*n* = 3 biological replicates). Asterisks indicate statistically significant differences between evaluated regions within the same treatment group, determined by two-way ANOVA followed by Tukey’s post hoc test: ns (not significant, *p* ≥ 0.05), * *p* < 0.05, ** *p* < 0.01, *** *p* < 0.001, **** *p* < 0.0001.

Given the critical role of *PPARγ-2* in regulating adipogenesis, we investigated how HDACis affect its expression through modulation of H3K9ac using a ChIP assay (Figure 5A). We designed different primers to analyze chromatin associated with the promoter region, spanning from −655 to +22 relative to the transcription start site (TSS), as well as a distal coding region of *PPARγ* located at +1586 relative to the TSS.

ChIP assays were performed on days 0 and 14 of adipogenic induction, corresponding to the time points with the highest levels of H3K9ac during differentiation. On day 0, significant differences in H3K9ac binding at *PPARγ-2* were observed among treatments with the epigenetic inhibitors VPA and TSA, as well as in the control group (cells cultured in adipogenic media). In the control group, H3K9ac enrichment was detected at site 3, while in VPA-treated cells, enrichment occurred at sites 2 and 4. Conversely, TSA-treated cells exhibited enrichment at sites 1 and 4 (Figure 5B). By day 14 of adipogenesis, sustained enrichment of H3K9ac was observed at site 1, likely within the *PPARγ-2* promoter region, across all treatments. Notably, H3K9ac enrichment at site 2 increased only in TSA-treated cells (Figure 5C). These findings indicate that the epigenetic inhibitors VPA and TSA promote an increase in H3K9ac levels, thereby enhancing chromatin accessibility and facilitating *PPARγ-2* gene transcription. Furthermore, the enrichment patterns observed at the various promoter sites correspond with acetylation peaks on key induction days, contributing to the early expression of *PPARγ-2* in the VPA-treated group. This underscores the crucial role of H3K9ac enrichment in the epigenetic regulation of adipogenesis and the activation of genes involved in cell differentiation.

## 3. Discussion

This study focused on the contribution of the euchromatin mark H3K9ac to the molecular program of adipogenesis in PDLSCs. In recent years, DSCs have emerged as a promising resource for regenerative medicine due to their biological characteristics and cellular plasticity. Consequently, understanding the regulatory mechanisms that govern the cellular decision-making processes of DSCs has become a topic of significant interest. Epigenetic regulation plays a vital role in stem cell differentiation, acting as a key driver of the transcriptional programming required for tissue specialization [34]. PDLSCs exhibit a limited capacity for adipogenic lineage commitment. It has been reported that DSCs exhibit variable responses to adipogenesis in vitro, with PDLSCs showing a greater propensity for adipogenic differentiation compared to DPSCs [21]. Regarding mesenchymal markers, the PDLSC population displayed higher expression levels of *CD73* transcripts, indicating a rich presence of MSCs. Additionally, the detection of pluripotency markers suggests that the PDLSC population possesses pluripotent differentiation potential, which is consistent with observations from cell differentiation assays (Figure 1).

VPA and TSA are widely used HDACis known for their roles in inhibiting cell proliferation, arresting cell cycle progression, and inducing cell differentiation and apoptosis [35]. In this study, PDLSCs were treated with these compounds for 72 h prior to adipogenic induction. The results demonstrated that these epigenetic inhibitors promote adipogenic differentiation in PDLSCs. Notably, we observed statistically significant differential effects between the inhibitors: TSA-treated cells exhibited increased lipid deposition, resulting in a higher density of preadipocytes in vitro compared to VPA-treated cells (Figure 2). Similar effects have been reported in studies investigating osteogenic differentiation in cells of dental origin. To date, however, there are no reports on the effects of these inhibitors on adipogenesis in human dental-derived cells, although their impacts have been described in other cell models. For example, continuous exposure to VPA (≥1.45 mM) and TSA (≥ 3 nM) in murine preadipocytes resulted in reduced lipid deposition [27]. In human preadipocytes and umbilical cord cells undergoing adipogenic induction, treatment with VPA at concentrations of 1 mM and 10 mM led to decreased intracellular lipid droplet formation compared to untreated cells. It is important to note that non-specific (pan-HDACs) inhibitors like VPA and TSA not only differentially modulate HDAC activity but may also affect non-transcriptional mechanisms, including apoptosis, cell cycle arrest, and differentiation [36]. These pleiotropic effects could explain the divergent outcomes observed across different cell types and experimental systems. For example, TSA enhances osteogenic differentiation in gingival mesenchymal stem cells [37]. In contrast, the same inhibitor suppresses self-renewal and reduces the efficiency of adipogenic, chondrogenic, and neurogenic differentiation in mesenchymal stem cells derived from umbilical cord and adipose tissue [38]. Therefore, further investigation is needed to determine whether combination or sequential treatment with these inhibitors might differentially regulate adipogenic differentiation potential.

Regarding adipogenesis, it is noteworthy that no other studies have provided a temporal morphological evaluation for comparison with the present work. To determine whether the changes observed during adipogenesis induced by VPA and TSA result not only from stimulation by the induction medium but also from inhibition of class I HDACs, we evaluated H3K9ac protein levels with and without inducers in the AIM. H3K9ac levels increased more with VPA treatment compared to TSA (Figure 3). These results suggest a positive effect of both VPA and TSA on H3K9ac. In both cases, the inhibitors had a greater effect when treatment was administered recently (Figure 3C). The favorable impact of H3K9ac modulation by HDACis on differentiation capacity is consistent with reports from various cellular models. In murine embryonic stem cells, treatment with 5 or 25 nM TSA resulted in increased H3K9ac levels after incubation periods of 0, 4, and 16 h, a pattern similarly observed with 0.5 mM VPA. In human cells, the regulatory effect of these inhibitors on differentiation efficiency has also been documented. For example, in PDLSCs, administration of VPA (0.1 or 0.5 mM) every 72 h enhanced the efficiency of differentiation toward osteogenesis [39]. In human embryonic cells, a temporal evaluation of neuronal differentiation at 0, 4, and 8 days under treatment with 10 ng/mL TSA showed increased levels of H3K9ac. This treatment had a dual effect on gene expression—repressing genes associated with pluripotency while promoting the expression of genes involved in neuronal commitment [40]. Although information on the effects of VPA and TSA during adipogenesis in PDLSCs is limited, our evaluation of the temporal acetylation profile provided significant insights. In cells treated with HDACis under adipogenic induction, acetylation peaks were detected at time points coinciding with observed morphological changes. This dynamic pattern of H3K9ac serves as a clear indicator of chromatin remodeling, potentially facilitating the expression of genes associated with the adipogenic phenotype. To test this hypothesis, we analyzed the expression of the adipogenic genes *PPARγ-2* and *C/EBPβ* during adipogenesis in the presence of HDACis. The results indicated that during the early stages of adipogenic differentiation, *C/EBPβ* levels increased in response to stimulation by the adipogenic differentiation cocktail. The *C/EBPβ* gene promotes the activation of master regulatory genes such as *PPARγ-2* and *C/EBPα* by binding to their promoters [41]. The elevated *C/EBPβ* levels observed in VPA-treated cells on day 7 may be associated with the increase in *PPARγ-2* levels, whereas in cells without inhibitors and those treated with TSA, *PPARγ-2* expression continued to increase significantly until day 14 (Figure 4).

Some studies have indicated that chromatin organization serves as a key regulatory mechanism in stem cell differentiation [42,43]. The regulation of global histone acetylation levels by epigenetic modulators such as HDACs is a powerful strategy to promote chromatin relaxation and, consequently, the selective activation of genes involved in differentiation. Therefore, it is reasonable to hypothesize that applying HDACis to PDLSCs during the early stages of differentiation could influence their commitment to a specific lineage. To explore this, we performed ChIP-qPCR analysis targeting genomic regions associated with *PPARγ-2* activation. H3K9ac enrichment was detected at several potential *PPARγ-2* binding sites, suggesting that treatment with VPA or TSA enhanced chromatin accessibility, leading to increased expression of *PPARγ-2* transcripts (Figure 5). This may explain the morphological differences observed in TSA-treated cells, where lipid droplets formed in greater quantity and with a smaller diameter compared to those in VPA-treated cells (Figure 2).

On the other hand, an increase in H3K9ac at the TSS has been widely associated with enhanced transcriptional activity [44]. For example, Paino et al. [42] reported that dental pulp stem cells undergoing osteogenic differentiation show increased acetylation of histone H3 (H3ac) in the promoter regions of the osteogenic genes osteocalcin (*OC*) and bone sialoprotein (*BSP*). Furthermore, it has been demonstrated that VPA enhances the hepatic differentiation of human bone marrow mesenchymal stem cells (BMMSCs) by increasing the acetylation of histones H3 and H4 [45]. In the field of adipogenesis, unlike differentiated cells, stem cells derived from adipose tissue exhibit an increase in global H3ac levels within 24 h of adipogenic induction [46]. Consistent with this finding, a positive correlation has been observed between the enrichment of H4K8ac and H4K12ac at promoter regions and the expression of adipogenic genes such as *C/EBP*, *PPARγ*, and adiponectin during adipocyte formation in porcine MSCs [30]. In line with these studies, our results suggest that chromatin remodeling may be triggered by an early stimulus mediated by HDACis, which could enhance the expression of HATs [32] and adipogenesis-related genes.

Furthermore, adipogenic differentiation is known to involve temporal and spatial changes in the expression of adipogenic genes. In this context, the differential levels of H3K9ac enrichment observed at the *PPARγ-2* promoter on days 0 and 14 of adipogenesis may result from HAT activity under HDAC inhibition, which could enhance euchromatin transcriptional potential and promote *PPARγ-2* expression in PDLSCs. Although these findings are suggestive, further validation is needed to confirm the functional relationship between H3K9ac enrichment and *PPARγ-2* transcriptional activation. Nonetheless, the results indicate that chromatin-modifying enzymes, such as HDACs acting on H3K9ac, play a crucial role in regulating adipogenesis in PDLSCs, potentially offering novel therapeutic targets for disorders involving aberrant fat cell generation.

## 4. Materials and Methods

### 4.1. Cell Culture

This study utilized cryopreserved PDLCs obtained from the third molar of a 13-year-old patient, with prior informed consent (Approval Number: CIE-06-2017). PDLCs were cultured in Eagle’s alpha-modified Minimum Essential Medium (α-MEM, Gibco, Grand Island, NY, USA) supplemented with 10% fetal bovine serum (FBS, Gibco, Grand Island, NY, USA and 1% Antibiotic-Antimycotic solution (penicillin/streptomycin; P/S, Gibco, Grand Island, NY, USA) at 37 °C in a humidified incubator with 5% CO_2_. For subsequent experiments, PDLCs in passages 5–6 were used.

### 4.2. Evaluation of the In Vitro Specialization of PDLCs

PDLCs at passage 6 were induced in vitro to undergo chondrogenic (14 days), osteogenic (14 days), and adipogenic (21 days) differentiation. Cells were seeded in 6-well plates at a density of 1 × 10^4^ cells per well and maintained for 48 h in supplemented α-MEM containing 10% FBS and 1% P/S). After this initial incubation, the culture medium was replaced with the specific induction medium corresponding to each type of differentiation. For chondrogenic differentiation, the StemPro Chondrogenic Differentiation Kit (Gibco; Invitrogen, Grand Island, NY, USA) was used according to the manufacturer’s instructions. After 14 days, to visualize mucopolysaccharides in the extracellular matrix, the cells were fixed with 4% paraformaldehyde (PFA) for 15 min at room temperature (RT) and subsequently stained with Alcian Blue 8GX (Sigma-Aldrich, St. Louis, MO, USA). Digital photographs were captured using an inverted microscope (TCM400, LABOMED, Los Angeles, CA, USA). For osteogenic differentiation, the induction medium consisted of α-MEM supplemented with 10% FBS, 1% P/S, 50 μM ascorbic acid, 10 mM β-glycerophosphate, and 10 nM dexamethasone (all purchased from Sigma-Aldrich, St. Louis, MO, USA). To identify calcium nodules and/or surface mineralization, cells were fixed with 4% PFA at RT after 14 days and stained with 2% Alizarin Red S (Sigma-Aldrich, St. Louis, MO, USA). For adipogenic differentiation, PDLCs were treated with AIM, consisting of α-MEM supplemented with 10% FBS and 1% P/S, along with 1.7 μM insulin, 1 μM dexamethasone, 500 μM 3-isobutyl-1-methylxanthine, and 60 μM indomethacin. The cells were maintained with medium changes three times per week. After 28 days, to detect intracellular lipid droplets, the cells were fixed with 4% PFA at RT and stained with 0.1% Oil Red O (Sigma-Aldrich, St. Louis, MO, USA) in propanol for 10 min. Morphological changes associated with each type of differentiation were evaluated using an inverted microscope (TCM400, LABOMED), and digital images were captured.

### 4.3. Effect of HDACis During Adipogenesis

PDLSCs were seeded in T25 flasks at a density of 3.5 × 10^4^ cells and maintained in α-MEM supplemented with 10% FBS and 1% P/S growth medium for 48 h. Subsequently, the growth medium was supplemented with either 8 mM VPA or 100 nM TSA (Sigma-Aldrich, St. Louis, MO, USA) and applied for 72 h. At the end of this exposure period, the medium was replaced with the AIM described above, with medium changes performed twice weekly for 28 days. Photographs were taken on days 0, 7, 14, and 28 of induction using an inverted microscope (TCM400, LABOMED, Los Angeles, CA, USA).

### 4.4. Total RNA Extraction and Quantitative RT-PCR

Total RNA was isolated from passage 6 PDLSCs using the Direct-zol RNA kit (Zymo Research, Irvine, CA, USA, following the manufacturer’s instructions. For cDNA synthesis, reverse transcription reactions were performed with 1 µg of total RNA using the PrimeScript RT–PCR Kit (Takara Biotechnology, Dalian, China), also according to the manufacturer’s protocol. Quantitative RT-PCR was conducted in triplicate using iTaq Universal SYBR Green Supermix (BIO-RAD, Hercules, CA, USA). The expression of pluripotency markers (*NANOG*, *OCT4*, *SOX2*, *KLF4*, *c-MYC*) and surface markers (*CD73*, *CD90*, *CD105*) was assessed using the Eco Real-Time PCR System (Illumina, San Diego, CA, USA) with Eco™ Real-Time PCR system qPCR Software v2.0.6.0 (Illumina, San Diego, CA, USA). For the analysis of adipogenic markers (*PPARγ-2* and *C/EBPβ*) at days 0, 7, 14, and 28 of adipogenic induction, the Rotor-Gene Q system (Qiagen, Hilden, Germany) was used, and data were analyzed with Rotor-Gene Q Series Software 2.3.5.1 (Qiagen, Hilden, Germany). The reaction mixture, prepared in a 200 μL microtube, contained 1 µL of cDNA (150 ng/µL), 5 µL of iTaq™ Universal SYBR Green Supermix (Bio-Rad), and 1 µL of a forward and reverse primer mixture (10 µM) specific to each target as described in Table 1. The final reaction volume was adjusted to 10 µL with ultrapure water (H_2_O-UP). The thermal cycling conditions were as follows: initial denaturation at 95 °C for 5 min, followed by 40 cycles of 95 °C for 40 s, and annealing at the temperature specified for each primer set (see Table 1) for 40 s. Gene expression changes were calculated relative to 18S rRNA using the 2^−ΔΔCT^ method [47] to measure the expression levels of pluripotency markers, surface markers, and adipogenic markers.

### 4.5. Western Blotting

PDLSCs subjected to adipogenic induction and pretreated with VPA (8 mM) or TSA (100 nM) for 72 h were lysed by ultrasonic shearing in RIPA buffer (Invitrogen) supplemented with a protease inhibitor cocktail (1:100) (Sigma-Aldrich). Protein extracts were collected on days 0, 7, 14, and 28 of adipogenic induction, and total protein content was quantified using the BCA assay (Bioscience, Hamburg, Germany). For each assay, 10 μg of total protein from the extracts was utilized. Western blot analysis was performed using primary antibodies against histone H3 (1:10,000) (Millipore Cat# 07-690, RRID: AB_417398, Burlington, MA, USA) and H3K9ac (1:2000) (Millipore Cat# 07-352, RRID: AB_310544), along with an HRP-conjugated anti-IgG secondary antibody (1:2500) (Millipore Cat# 31460, RRID: AB_228341). Proteins were visualized using the ECL system according to the manufacturer’s instructions and exposed to X-ray film (Amersham Hyperfilm™ ECL, Amersham, UK). Quantitative analysis was performed using ImageJ software (Version 1.54, RRID: SCR_003070).

### 4.6. Chromatin Immunoprecipitation Followed by Quantitative Polymerase Chain Reaction

PDLSCs pretreated with 8 mM VPA or 100 nM TSA during adipogenic induction on days 0 and 14 were collected by digestion with 0.25% Trypsin-EDTA (Gibco™, Grand Island, NY, USA). The resulting cell suspension was fixed with 1% (*v*/*v*) formaldehyde for 10 min to cross-link protein-DNA interactions and then quenched with 1.25 M glycine. Cells were lysed using a high-salt buffer (0.1% SDS, 1% Triton X-100, 2 mM EDTA, 20 mM Tris-HCl pH 8, 500 mM NaCl), LiCl buffer (250 mM LiCl, 1% NP-40, 1% sodium deoxycholate, 1 mM EDTA, 10 mM Tris-HCl pH 8.1), and subjected to ultrasonication (CV18, Cole Parmer, Vernon Hills, IL, USA) for 30 cycles (30 s on/off) at 50% amplitude, generating DNA fragments ranging from 100 to 500 bp. The lysate was then diluted 10-fold with dilution buffer and pre-cleared with protein G-agarose (Invitrogen) at 4 °C for 1 h. Chromatin was immunoprecipitated using a 1:20 dilution of H3K9ac antibody (Invitrogen Cat# 49-1009, RRID: AB_2533860) and incubated overnight at 4 °C. Following incubation, 50% protein G-agarose was added, and samples were shaken at 4 °C for 3 h. The immune complexes were sequentially washed with the following buffers: low-salt buffer (1% SDS, 1% Triton X-100, 2 mM EDTA, 20 mM Tris-HCl pH 8, 150 mM NaCl), high-salt buffer, LiCl buffer (250 mM LiCl, 1% NP-40, 1% sodium deoxycholate, 1 mM EDTA, 10 mM Tris-HCl pH 8.1), and TE buffer (1 mM EDTA, 10 mM Tris-HCl pH 8.1). The DNA-protein complexes were then eluted using 1% (*w*/*v*) SDS and 100 mM NaHCO_3_ buffer, followed by precipitation with 200 mM NaCl and overnight incubation at 65 °C. The following day, the sample was incubated at 37 °C with RNase for 30 min, followed by digestion with proteinase K (20 μg/mL) in 10 mM EDTA and 35 mM Tris-HCl, pH 6.8. DNA purification was carried out using phenol/chloroform/isoamyl alcohol (25:24:1) extraction. The supernatant was collected and mixed with 3 M sodium acetate (pH 5.2) and 1 μg of glycogen per volume of supernatant, then precipitated with 96% ethanol at −20 °C overnight. The resulting pellet was washed with 70% ethanol, air-dried at RT, and resuspended in 1X TE buffer. The final purified DNA was stored at −80 °C. ChIP-qPCR was performed using primers targeting regions upstream and downstream of the *PPARγ-2* TSS (Table 1). ChIP-qPCR data are presented as a percentage of input, calculated according to the method described by Solomon et al. [48] using the formula: $\%\;Input=2^{\left(cq(IN)\;-\;{Log}_{2}(Dfv)\;-\;cq(IP)\right)}\times 100$.

### 4.7. Statistical Analysis

Data are expressed as mean ± standard deviation from three independent experiments performed in triplicate. Where appropriate, data were analyzed by one-way ANOVA followed by Dunnett’s or Tukey’s post-hoc test using GraphPad Prism version 10.1.1 software. Statistical significance is indicated as * (*p* < 0.05), ** (*p* < 0.01), *** (*p* < 0.001), and **** (*p* < 0.0001).

## 5. Conclusions

**Figure 6:**
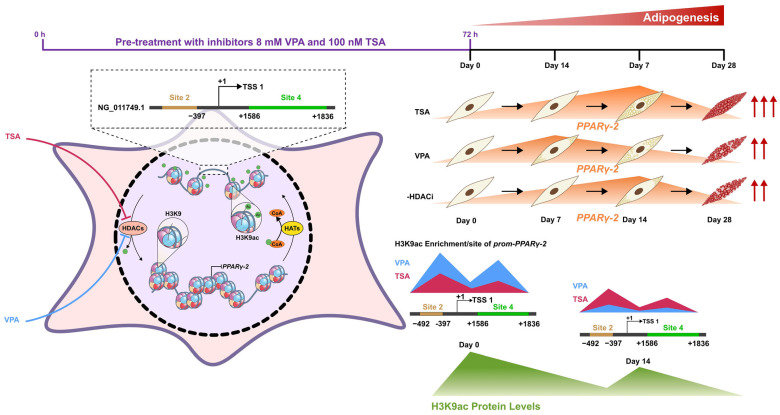
Proposed mechanism of H3K9 acetylation-mediated regulation of adipogenesis in periodontal ligament stem cells: The lipophilic histone deacetylase inhibitors VPA (144 Da) and TSA (302.37 Da) passively diffuse across the plasma membrane and accumulate in the nucleus, where they inhibit class I/II histone deacetylases. This inhibition increases acetylation of histone H3 at lysine 9 (H3K9ac), promoting chromatin relaxation at the *PPARγ-2* promoter region (NG_011749.1). The resulting open chromatin structure facilitates recruitment of the transcriptional machinery, enhancing PPARγ-2 expression (orange peaks). Our results reveal distinct temporal acetylation patterns between the two treatments. VPA-treated cells exhibit an immediate peak in H3K9 acetylation ( blue peaks) following treatment (day 0 of adipogenesis), leading to early activation of *PPARγ-2* transcription, and accelerated morphological changes. This early effect is reflected by pronounced lipid droplet accumulation during the initial stages of differentiation. In contrast, TSA-treated cells display a more sustained acetylation profile (red peaks), with a progressive accumulation of H3K9ac marks (green peaks) at the *PPARγ-2* promoter throughout the differentiation process. This epigenetic pattern corresponds to prolonged gene expression and more gradual morphological changes but ultimately results in higher preadipocyte density and more abundant lipid accumulation at later stages, as confirmed by specific in vitro culture staining. The black line represents the *PPARγ-2* region of the sequence used for primers design, with site 2 indicated by the brown line and site 4 by the green line.

In summary, this study analyzed changes in H3K9 acetylation induced by class I HDACis (TSA and VPA) during the adipogenic differentiation of PDLSCs. Our observations demonstrate that TSA and VPA promote adipogenesis in PDLSCs via a mechanism dependent on H3K9 hyperacetylation (Figure 6). The key findings include: (1) significant enrichment of H3K9ac at the *PPARγ-2* promoter, particularly in TSA-treated cells, which correlates with increased lipid droplet formation; and (2) distinct temporal expression patterns of adipogenic genes (*C/EBPβ* and *PPARγ-2*) in response to TSA and VPA treatments. These results indicate that chromatin remodeling mediated by H3K9ac is a critical regulator of adipogenic commitment in PDLSCs, with effects that vary depending on the specific inhibitor used. However, this study has important limitations. First, the analysis focused exclusively on H3K9ac, leaving the potential contributions of other epigenetic marks unexplored. Second, the precise mechanisms linking specific acetylation at the *PPARγ-2* locus to transcriptional activation remain to be elucidated. Future research should focus on: (i) comprehensive epigenomic profiling—including DNA methylation and additional histone modifications—during PDLSC adipogenesis; (ii) validation of these findings through functional assays assessing adipocyte metabolism; and (iii) developing strategies to selectively modulate acetylation at specific loci via epigenetic editing. These advances could provide a foundation for translational applications in adipose tissue engineering and the treatment of metabolic disorders, capitalizing on the accessibility and plasticity of dental stem cells.

## Figures and Tables

**Table 1:** Primers sequences used for amplification of molecular markers of interest.

M.M.	DIRECT SEQUENCE 5’→3’	REVERSE SEQUENCE 5’→3’	FS (pb)	AT (°C)	Ref.
**Stemness markers**					
*Oct4*	GAAAGGGACCGAGGAGTA3	CCGAGTGTGGTTCTGTAAC	196	62	[40]
*NANOG*	TGCTGAGATGCCTCACACGGA	TGACCGGGACCTTGTCTTCCTT	117	60	
*SOX2*	ACACCATECCATCCACACT	CCTCCCCAGGTTTTCTGT	117	62	[41]
*KLF4*	TACCAAGAGCTCATGCCACC	CGCCTAATCACAAGTGTGGG	114	60	
*c-MYC*	GGACCCGCTTCTCTGAAAGG	TAACGTTGAGGGGCATCGTC	104	60	
**Mesenchymal surface markers**					
*CD73*	CTCAAGACCAGGAAGTCCATA	GATGAGGAAGGCACCAAAG	131	58	
*CD90*	GTCCTCTACTTATCCGCCTTC	GACCAGTTTGTCTCTGAGCAC	123	56	[42]
*CD105*	CAGCATTCCTGAAGATCCAAG	GATTGAGAGGAGCCATCCAG	120	56	
**Adipogenic markers**					
*PPARγ-2*	CAGTGGGGGCTCATAA	5′CTTTTGGCATACTCTGTGAT	137	60	[43]
*C/EBPβ*	CACAGCGACGACTCAAGATCC	GGAGTACTIGCGCTCAGGAGGAGC	188	58	[44]
**Housekeeping gene**					
*18s*	GGACAGGATTGACAGATTGAT	AGTCTCGTTCGTTATCGGAAT	111	60	Own
**ChIP-qPCR assay (Promoter *PPARγ2*)**					
*Site 1*	AGTGCAGTGGTGTGATCTCA	GATTACAGGCGTGCTACCAC	105	63.5	Own
*Site 2*	GTCTCGAACTCCTGACCTCA	AAGGTATACAGGCCAGGCAC	95	63.5	
*Site 3*	GCGCCCAGATGAGATTACTT	AGAATGGCATCTCTGTGTCAA	148	63.5	
*Site 4*	CCTCTCACATGTCTCCATACACA	CTGAAATGAAATAATAAAGTTTCAACA	250	63.5	

FS: Fragment size in base pairs (bp). AT: Alignment temperature. Ref: Reference.

## Data Availability

All data are available within this manuscript.
